# Evaluating Computer Vision, Large Language, and Genome-Wide Association Models in a Limited Sized Patient Cohort for Pre-Operative Risk Stratification in Adult Spinal Deformity Surgery

**DOI:** 10.3390/jcm13030656

**Published:** 2024-01-23

**Authors:** Ethan Schonfeld, Aaradhya Pant, Aaryan Shah, Sina Sadeghzadeh, Dhiraj Pangal, Adrian Rodrigues, Kelly Yoo, Neelan Marianayagam, Ghani Haider, Anand Veeravagu

**Affiliations:** 1 Stanford University School of Medicine, Stanford University, Stanford, CA 94304, USA; rdpant@stanford.edu (A.P.); sinas@stanford.edu (S.S.); 2 Department of Computer Science, Stanford University, Stanford, CA 94304, USA; aaryans@stanford.edu; 3 Department of Neurosurgery, Stanford University School of Medicine, Stanford University, Stanford, CA 94304, USA; pangal@stanford.edu (D.P.); kellyyoo@stanford.edu (K.Y.); njm39@stanford.edu (N.M.); ghanih@stanford.edu (G.H.); anand.veeravagu@stanford.edu (A.V.); 4 Department of Neurosurgery, Massachusetts General Hospital, Boston, MA 02114, USA; arodrigues11@mgb.org

**Keywords:** adult spinal deformity, computer vision, large language model, genome wide associated study, sepsis, neurological complication, spine surgery

## Abstract

**Background**: Adult spinal deformities (ASD) are varied spinal abnormalities, often necessitating surgical intervention when associated with pain, worsening deformity, or worsening function. Predicting post-operative complications and revision surgery is critical for surgical planning and patient counseling. Due to the relatively small number of cases of ASD surgery, machine learning applications have been limited to traditional models (e.g., logistic regression or standard neural networks) and coarse clinical variables. We present the novel application of advanced models (CNN, LLM, GWAS) using complex data types (radiographs, clinical notes, genomics) for ASD outcome prediction. **Methods:** We developed a CNN trained on 209 ASD patients (1549 radiographs) from the Stanford Research Repository, a CNN pre-trained on VinDr-SpineXR (10,468 spine radiographs), and an LLM using free-text clinical notes from the same 209 patients, trained via Gatortron. Additionally, we conducted a GWAS using the UK Biobank, contrasting 540 surgical ASD patients with 7355 non-surgical ASD patients. **Results:** The LLM notably outperformed the CNN in predicting pulmonary complications (F1: 0.545 vs. 0.2881), neurological complications (F1: 0.250 vs. 0.224), and sepsis (F1: 0.382 vs. 0.132). The pre-trained CNN showed improved sepsis prediction (AUC: 0.638 vs. 0.534) but reduced performance for neurological complication prediction (AUC: 0.545 vs. 0.619). The LLM demonstrated high specificity (0.946) and positive predictive value (0.467) for neurological complications. The GWAS identified 21 significant (*p* < 10^−5^) SNPs associated with ASD surgery risk (OR: mean: 3.17, SD: 1.92, median: 2.78), with the highest odds ratio (8.06) for the LDB2 gene, which is implicated in ectoderm differentiation. **Conclusions:** This study exemplifies the innovative application of cutting-edge models to forecast outcomes in ASD, underscoring the utility of complex data in outcome prediction for neurosurgical conditions. It demonstrates the promise of genetic models when identifying surgical risks and supports the integration of complex machine learning tools for informed surgical decision-making in ASD.

## 1. Introduction

Adult spinal deformity (ASD) is a condition characterized by abnormality in the three dimensional structure of the spine, most commonly the thoroco–lumbar segments [1]. Such deformities include scoliosis, kyphosis, and lordosis, where lumbar lordotic reduction is a significant driver of ASD [1]. Some specific deformities are more pathologic than others, and result in a reduction in quality of life (QOL) and increased disability [2]. ASD prevalence increases with age, with a general prevalence of 2–32% and a prevalence of 30–68% in the elderly population [3,4]. Surgery may correct the deformity; however, these surgeries are complex, with neurologic complications reported at 1–10% and non-neurologic complications reported at as much as 50% [3]. Elderly patients have an increased complication rate, likely due to diminished physiological reserve and greater frailty, but may gain greater QOL improvement from surgical correction, highlighting the complicated decision for ASD surgical intervention [4,5].

Surgical decision making for ASD correction is multifactorial, considering frailty, comorbidities, symptomatology, and degree of deformity [6]. These multi-factorial considerations underscore the need for accurate pre-operative risk stratification, which is challenging in ASD patients. To determine which patients would benefit from corrective surgery for ASD, traditional machine learning approaches (i.e., decision trees, nomograms, logistic regression, neural network) have been applied to predict outcomes such as complication, reoperation, readmission, and QOL metrics. Traditional approaches in ASD surgical risk stratification and existing predictive models are limited by the following three elements: (i) small patient cohorts due to the condition’s relatively small incidence and prevalence (e.g., a prior prediction of QOL outcomes used only 191 patients) [7], (ii) limited data types included as predictive variables (i.e., demographic, clinical, radiographic, or operative variables), and (iii) utilization of traditional machine learning models rather than state-of-the-art advanced models. While some of these models have predicted outcomes including major complication (AUC of 0.89) [8] and health-related QOL (c-statistic of 0.739) [7], the validation of web-based predictive models for major complication, reoperation, and readmission in ASD using a cohort of adult symptomatic lumbar scoliosis patients found an AUC of only 0.6 for all outcomes, demonstrating that current feature-based models fail to accurately risk-stratify patients [9]. The poor performance of these traditional models in ASD outcome prediction motivates the need to survey the performance of state-of-the-art models.

Currently, there are advanced models, including computer vision and natural language processing (NLP) models (including large language models (LLMs)), that have been pre-trained on thousands to millions of patients and which can subsequently be fine-tuned for specific outcomes with smaller patient sets. We hypothesized that such pre-trained advanced models may be leveraged to address all three limitations in ASD surgical risk stratification, therefore improving surgical decision making. Therefore, in this work we investigate the application of advanced models, including computer vision, natural language processing, and genome wide association study (GWAS), for ASD risk stratification.

## 2. Methods

### 2.1. Inclusion and Exclusion Criteria

Adult patients, from 1 January 2016 to 26 June 2023, who underwent spine surgery to address ASD were included in the study. A procedure was determined as a spine surgery intervention for ASD if it involved any one of the following current procedural terminology (CPT) codes: 22800, 22802, or 22804. Patients younger than 18 were excluded.

### 2.2. Data Source

The Stanford Research Repository was used to identify patients who met the inclusion criteria. The patient population included patients with surgeries performed by any spine surgeon at Stanford University Hospital. ASD patients who experienced post-operative complications had a higher mean age (entire cohort: 38.3, pulmonary complication: 41.1, delirium: 68.7, neurological complication: 54.8, sepsis: 43.1).

### 2.3. Variables and Outcomes

All X-ray imaging data, demographics, laboratory results, diagnostic and procedural codes, and clinical and procedural narrative reports were collected for each patient. There was variation in the word count of pre-operative clinical notes (mean: 808.3, SD: 564.6, min: 16, max: 2976). The surgical age of each patient was determined, and the most recent clinical notes and XR images were gathered and then manually screened for appropriate ASD-related notes and images. Clinical notes were deemed appropriate for the criteria if they were written by an attending surgeon and prior to the date of surgery. Therefore, despite prior demonstration of LLMs using operative notes [10], we did not include operative notes for the following two reasons: (1) they may include complication information, thereby biasing the model, and (2) the purpose of our model would be for improved patient counseling and selection, meaning that use of an operative note as input would not be appropriate. Primary outcomes for the study were selected based on the clinical consideration of frequency, predictive value, and data availability. These primary outcomes were the prediction of a pulmonary complication, neurological complication, sepsis, and delirium within 90 days of the index ASD surgery. These were selected based on the availability of sufficient positive instances for reliable model training. Other outcomes extracted were three-month revision, two-year revision, five-year revision, 90-days post-operative; altered mental status, confusion, mortality, and patient-reported outcome measures (PROMs); and PHQ2, PHQ9, ODI, and SRS. Due to the very low frequency of positive examples for these additional outcomes, models were only trained for the prediction of the primary outcomes.

### 2.4. Data Preprocessing

#### 2.4.1. CNN

A CNN was trained and tested for the prediction of specific post-operative complications (pulmonary complication, neurological complication, sepsis, and delirium) in ASD surgery using pre-operative spine radiographs as input. A total of 1549 radiographs from 209 patients surgically treated for ASD were sourced from the Stanford Research Repository. For training and testing, we employed a 70–30 split via random allocation. Stratification was undertaken at the patient level to ensure that all radiographs from a single patient were allocated to either the training or testing set, preserving patient-level integrity in the data. Radiographs were extracted and processed in PNG format. All training images were scaled to 224 × 224 pixels and normalized by the mean and standard deviation of images. Only pre-operative radiographs were used for outcome prediction.

#### 2.4.2. NLP

An LLM was developed and tested for the prediction of specific post-operative complications (pulmonary complication, neurological complication, sepsis, and delirium) in ASD surgery using pre-operative clinical notes as input. We extracted and utilized free-text clinical notes from the 209 patients, applying a 70–30 split for training and testing analogous to the CNN data preprocessing. We employed a large language model (LLM) (Gatortron) that has been extensively pre-trained on clinical notes by the University of Florida covering more than two million patients [11]. Patient-level stratification was similarly enforced to maintain consistency across all notes from an individual patient. Reports were truncated to 512 words as standard input for Gatortron.

### 2.5. Model Training and Evaluation

#### 2.5.1. CNN

To evaluate the effect of pre-training the CNN, two separate models were developed, one of which was without any pre-training and used only our ASD patient radiographs. We trained two distinct CNNs, the first was trained from scratch using our dataset of ASD radiographs and the second using a pre-trained, publicly available CNN from the VinDr-SpineXR database [12] and which was subsequently fine-tuning the model on our ASD dataset. The first 50 layers of the pre-trained model were frozen prior to fine-tuning. For each of the four clinical outcomes, a 70–30 training and validation split was used. Training used the stochastic gradient descent (SGD) optimizer, with an initial learning rate of 0.01 and batch size of 32. The validation set was used to determine epoch hyperparameter. The CNN trained from scratch was trained to the following epochs (pulmonary complication: 979, neurological complication: 619, sepsis: 199, delirium: 159), while the pre-trained CNN was fine-tuned to the following additional epochs (pulmonary complication: 519, neurological complication: 39, sepsis: 279, delirium: 19).

#### 2.5.2. NLP

Due to the large data requirement associated with training a large language model, only one LLM was developed, which used the pre-trained Gatortron LLM model. A 70–30 training and validation split was similarly used. Training utilized the AdamW optimizer, with a learning rate of 1e-6, a batch size of 16, and L2 regularization set at 0.01. The LLM was fine-tuned to the following epochs (pulmonary complication: 4, neurological complication: 20, sepsis: 5, delirium: 3). Fine-tuning began to overfit after 10 epochs across all outcomes (Supplemental Figure S1).

#### 2.5.3. Performance Metrics

To assess both the CNN and LLM, we calculated the AUC, F1 score, sensitivity, specificity, and positive predictive value (PPV).

### 2.6. Genome-Wide Association Study (GWAS)

#### 2.6.1. Data Source

A GWAS was performed to identify genomic markers for ASD patients who required spine surgical intervention. Using the UK Biobank (N = 502,364), a cohort of patients who had undergone spine surgical intervention for ASD was defined (N = 540), as was a cohort of patients who had not undergone any spine surgical intervention but did have an ASD diagnosis (N = 7355). This outcome phenotype was selected due to the large (>500 patients) cohort available in the UK Biobank. Currently there is not a large cohort of ASD patients who have a specific post-operative complication available in the UK Biobank.

#### 2.6.2. Inclusion and Exclusion Criteria

Both cohorts were restricted to subjects 18 years or older, with exclusion criteria of any diagnosis of a malignant neoplasm of the spinal cord, cauda equina, or a benign neoplasm of the spinal cord. The surgical cohort was defined so as to include any participants that had ever had an instrumented correction of deformity of their spine or other operative procedural correction of deformity of their spine, and to exclude any participants that had ever had an extirpation of a lesion of the spine, decompression of a fracture of the spine, other reduction of a fracture of the spine, or a fixation of a fracture of their spine. These exclusion criteria were employed so as to restrict cohorts to elective procedures and remove trauma as a third variable that may influence the need for operative correction of the ASD.

#### 2.6.3. Phenotype Preprocessing

Quality control was run for subject phenotypes (i.e., ASD surgery, ASD no surgery). The standard UK Biobank filtering method was used and was restricted to the following: sex and genetic sex are the same, white British ancestry, no sex chromosome aneuploidy, no kinship found. Due to the UK Biobank’s limited heterogeneity of racial identities, the standard filtering includes a restriction to those of white British ancestry in order to eliminate potential third variables associated with the inclusion of a small set of other races while attempting to identify phenotype-specific genomic factors that should later be validated across all racial groups. After phenotype quality control, the ASD surgery group had 268 subjects, whereas the ASD no surgery group had 3803 subjects. There were 469,835 samples, where 254,616 were female and 215,156 were male.

#### 2.6.4. Genotype Preprocessing

PLINK was used to perform quality control and filter the whole exome sequencing (WES) data, standard parameters were selected (minor allele frequencies (MAF): 0.0005, minor allele counts (MAC) = 20, missing call rates per variant (geno) = 0.1, missing call rates per sample (mind) = 0.1, minor allele maximum frequency (max-maf) = 0.9995). For each chromosome, a list of single nucleotide polymorphisms (SNPs) and WES ids that passed the filter were collected.

#### 2.6.5. WES Association Study

Samples, variants, and phenotypes that passed the above quality control were used for the associated study. A logistic regression was run using PLINK2 on WES data for each chromosome. Sex and age were marked as covariates. SNPs were mapped to their genes, functional consequence, and clinical significance (if known) using the dbSNP database of the National Library of Medicine (NLM) National Center for Biotechnology Information (NCBI).

### 2.7. Ethical Considerations

This study was performed under IRB #69667 at Stanford University.

## 3. Results

### 3.1. Computer Vision Prediction of Surgical Outcomes Using Radiographs

A CNN was trained on pre-operative spine radiographs to predict the following four post-operative complications: pulmonary complications, neurological complications, sepsis, and delirium. To establish a baseline level of performance without transfer learning, we trained the CNN from scratch using our ASD dataset (Table 1). While the average AUC across outcomes (0.596) is low, an average specificity of 0.815 was achieved. The prediction of pulmonary complication was particularly successful (specificity: 0.822, PPV: 0.235, F1: 0.288).

**Figure 1:**
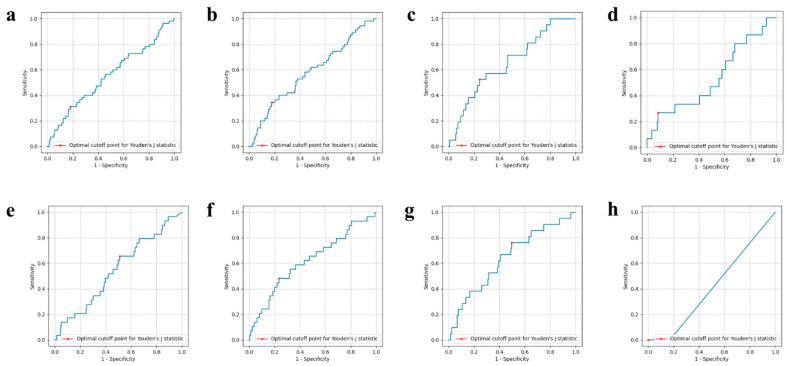
Receiver operating characteristic (ROC) curves for (**a**–**d**) CNN trained from scratch, and (**e**–**h**) fine tuning VinDr-SpineXR CNN on each outcome. The optimal cutoff threshold point (Youden’s J statistic) is represented by a red point on each curve. Each model was trained independently for (**a**,**e**) pulmonary complications, (**b**,**f**) neurological complications, (**c**,**g**) sepsis, and (**d**,**h**) delirium.

The effects of using the pre-trained VinDr-SpineXR CNN with fine-tuning on the ASD patient cohort were surprising in this rare condition setting. The predictive power of the model was approximately similar for some outcomes (i.e., pulmonary complication), markedly lower for other complications (i.e., neurological complications and delirium), and markedly higher for sepsis prediction. In the case of sepsis prediction, large improvements in sensitivity (0.713 vs. 0.198) and AUC (0.638 vs. 0.534) of the model were achieved compared with the CNN without pre-training (Table 1). ROC curves for each model contrast the CNN trained from scratch with the CNN first pre-trained on VinDr-SpineXR (Figure 1).

### 3.2. Large Language Model (LLM) Prediction of Surgical Outcomes Using Clinical Pre-Operative Notes

A pre-trained LLM, Gatortron, was fine-tuned on the ASD cohort (Table 2). While the LLM achieved a low mean AUC across predicted outcomes (0.547), for the outcome with the greatest positive class frequency, pulmonary complication, it achieved successful performance across metrics (PPV: 0.410, F1: 0.545). Compared with the F1 achieved by the CNN for sepsis prediction (F1: 0.135), the LLM achieved evident improvement (F1: 0.383). Training and validation loss curves are given in Supplemental Figure S1.

### 3.3. GWAS for Identification of Loci Associated with Surgical Intervention for ASD Patients

**Figure 2:**
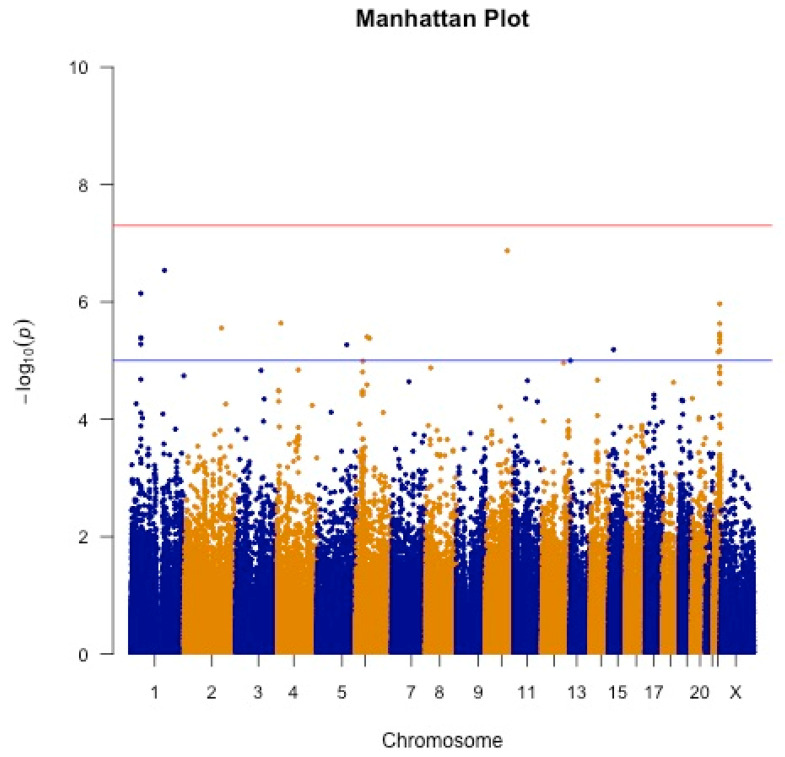
Manhattan Plot demonstrating 22 SNPs above a 1 × 10^−5^ threshold. Significant SNPs are spread across most chromosomes, with a high frequency of SNPs occurring on chromosomes 1 and 22. The blue line corresponds to a *p*-value above a 1 × 10^−5^ threshold. The red line corresponds to a *p*-value above a 5 × 10^−8^ threshold.

The patient cohort used for GWAS is derived from UK Biobank and distinct from the cohort used for CNN and LLM model development. A total of 21 SNPs were identified as significant using a 10-05 threshold in their association across a cohort of ASD patients who had undergone a surgical correction of their deformity and a cohort of ASD patients who had never undergone surgical correction (Figure 2, Table 3). The odds ratios (OR) for these SNPs ranged from 1.58 to 8.06, with a mean OR of approximately 3.17 and a median of 2.78, indicating variable but generally increased odds of the post-surgical state with these variants.

Analysis of the genomic distribution of these SNPs revealed involvement across 11 unique genes, with CELSR1 containing the highest number of significant SNPs (*n* = 8), followed by SLC6A9 (*n* = 4). The remaining genes each had one SNP associated with them. The functional consequences of these SNPs were predominantly intronic (*n* = 9), but also included missense variants and variants affecting gene expression regulation such as upstream and downstream transcript variants. Some SNPs were associated with more than one functional consequence, reflecting the complex and often unknown architecture of these genomic regions.

## 4. Discussion

This study demonstrates the effectiveness of cutting-edge ML models in forecasting post-surgical outcomes in ASD, highlighting the utility of complex data (patient imaging, clinical notes) for outcome prediction in neurosurgery. The recent external validation of ASD surgical outcome prediction models, which found poor performance (AUCs ~ 0.60) across complication, readmission, and reoperation outcomes, demonstrates that the current strategy for developing these models is insufficient to achieve clinically impactful machine learning decision-making support [9]. This strategy relies on standard predictive factors (demographic, surgical, radiographic) and traditional machine learning model types to leverage the small amounts of available data to predict outcomes. However, beyond the demonstrated poor predictive performance of this approach, there are clinical limitations. Firstly, models require manual input and become cumbersome and uninterpretable to the physician when including a large set of features [13,14], while, secondly, the models rely on predefined features which likely do not capture all relevant information [15]. To address these limitations, computer vision and natural language processing (NLP) offer the opportunity to use patient imaging and clinical notes, respectively, as inputs for a next-generation schema of spine surgery outcome prediction.

Computer vision and NLP have been identified as potentially powerful tools for surgical outcome prediction in the spine domain [16,17]. However, as of 2022, there were no computer vision tools [18] or NLP tools [19] widely used for diagnostics in spine surgery. The utilization of outdated models with low complexity has been identified as a limitation prohibiting clinical use [19]. The large amount of data needed to train these advanced models have restricted their development. However, advances in NLP [11] and computer vision [12] model pre-training on foundational clinical information allow for their application to data-limited domains such as ASD outcome prediction. In this paper, we demonstrate the success of computer vision, large language model (LLM), and genome-wide association studies (GWAS) in a limited ASD patient cohort for outcome prediction.

Both our CNN and NLP models achieve performance metrics comparable to the performance of the state-of-the-art models used for ASD outcome prediction [9] (Table 1 and Table 2). The highest performance was achieved by the fine-tuned LLM on pulmonary complication and sepsis outcomes, with sensitivity, specificity, and F1 scores showing the potential for downstream clinical utilization. The LLM outperformed the CNN for prediction of pulmonary, neurological, and sepsis outcomes. This observed performance difference may be attributed to the fact that the LLM was pre-trained on a much greater extent of data as part of Gatortron development. However, the performance difference may show the medical reasoning capacity of the LLM and how it is a more powerful model for outcome prediction. The use of advanced AI models offers several advantages in clinical practice. CNNs excel in image data analysis, such as MRI and CT scans, which are crucial in spine surgery. They can automate and enhance the accuracy of medical image interpretation, offering precise detection of spinal conditions [20,21] and of features that are too subtle for the human eye [22]. NLPs can extract insights from unstructured textual data, lowering the workload necessary to manually extract features, and can aid in rapid predictive modeling [19]. Analysis of free text reports have been used to detect outcomes such as incidental durotomies [23], intraoperative vascular injury in anterior lumbar spine surgery [24], postoperative wound infection after lumbar discectomy [25], and readmission after a posterior lumbar fusion [26]. Furthermore, CNN and NLP applications in spine surgery can streamline administrative tasks and optimize the allocation of resources, which can greatly enhance healthcare delivery. Finally, imaging data and clinical notes can be combined into a hybrid model to aid in preoperative planning and postoperative care, allowing neurosurgeons to tailor surgical approaches to individual patient needs [13].

Despite using a limited number of ASD patients who had undergone surgical intervention (N < 300), a GWAS identified 21 novel SNPs that differentiate ASD patients into a cohort which require surgical intervention, representing not only a novel research direction but also what we believe to be the first GWAS on a spine surgery utilization outcome. While the potential of GWAS for neurological disorders is known, the challenges of large required sample sets and resource requirements have limited its use in spine surgery research [27]. Our results show that GWAS designs can uncover SNPs for clinical risk stratification, patient counseling, and pathology forecasting (which may allow for earlier surgical decision making), even in rare spinal pathologies with limited numbers of patients. There are many factors which lead to the development and progression of spinal deformity and understanding specific genetic markers can differentiate which patients should receive intervention. CELSR1 (Table 3) was identified by the GWAS as a significant risk factor and a coding sequence variant in ASD patients who underwent surgical intervention. CELSR1, which has been previously implicated in neural tube defects [28], has been demonstrated to play a neuroprotective effect through neurogenesis promotion in cerebral ischemic injury through the Wnt signaling pathway [29]. These results suggest a potential role of CELSR1 to mediate neural functioning in spinal deformity, where its deficit leads to pathology. The identified risk genes (PDCD11, CDAN1, SLC6A9, MSTO1, CACNA1I, CELSR1, LNPK, LDB2, BRD8, FBXO9, EYS) should be further developed for the clinical stratification of ASD patients to allow for the earlier identification of appropriate surgical candidates before further (and often non-recoverable) deficits develop. The identified risk genes are for the prediction of spine surgery intervention in ASD, and not directly predictive of a complication following such intervention. However, the clinical application of these risk genes can allow for earlier intervention, which may improve outcomes and reduce complications in ASD surgery before the pathology and symptoms advance. This work focused on the intervention as an outcome due to the currently limited number of complications following ASD surgery in the UK Biobank. However, as this data availability grows, future work, motivated by our successful demonstration of GWAS in an ASD cohort, can develop GWAS to identify risk genes for specific complications and outcomes of ASD surgery.

This work introduces models that use imaging, clinical notes, and patient genomics to predict outcomes, including post-operative complications and the need for spine surgery intervention. Moreso, we show the potential of computer vision, large language models, and genome-wide associated models to interface with patient imaging, clinical notes, and genomics to predict relevant outcomes. Here we consider a potential workflow following the external validation and robust development of the models presented in this work to detail the clinical actionability of such models. When a patient presents with ASD, one of the first questions that a surgeon must consider is whether this patient is a surgical candidate. The patient may present with pathology that may not be intervened on, but that the surgeon worries will develop to cause non-reversible deficits. Such deficits may include structural worsening that would change the surgery type to a procedure with greater risk or might result in the compression of the spinal cord or the exiting of spinal nerves that may result in non-recoverable function or pain. If such factors appear clinically relevant, the surgeon may genotype the identified set of SNPs that have been found to be markers for an ASD patient who will eventually need to undergo a spine surgery intervention. Therefore, if the patient is positive for these markers, their pathology may be likely to worsen, supporting the decision for earlier surgery. However, the decision for surgery is further determined by considering the potential for a patient to undergo a major post-operative complication. Using already available pre-operative imaging and pre-operative clinical notes, the CNN and NLP would be utilized for the prediction of such complications. The surgeon would then use these three models to support their decision making regarding the appropriateness of a surgical intervention, enriched with an understanding of personalized surgical risks and risk genes informing whether the patient’s deformity is likely to develop to necessitate surgical intervention. To optimize the application of the models, future research should focus on broadening the spectrum of clinical inputs, enhancing the model’s ability to forecast a wider array of actionable clinical outcomes. This expansion would refine the decision-making process. Additionally, future work should aim to integrate these models, leveraging the collective strength of genomic, imaging, and clinical data. Such integration promises a streamlined surgical workflow and a more robust decision support system that considers all facets of patient information.

There are several limitations to the study. First, due to the limited size of the cohorts, certain outcomes had too few positive examples for training. Second, these models were fine-tuned on patient data from a single institution and therefore require external validation. Such external validation, as for all clinical model development, should include diverse patient data across regions, race, and comorbidities. This is a limitation frequently faced by predictive models in spine surgery; however, our fine-tuning approach mollifies this limitation by using models that were pre-trained on large patient sets from other institutions. Therefore, it is likely that these fine-tuned models may perform better on external patient data than traditional models trained on single institution data. Furthermore, while this work sought to evaluate the efficacy of NLP and CNN models for outcome prediction, the final robust models for clinical use may benefit from the inclusion of other predictive variables (e.g., surgeon experience). Finally, the GWAS used the UK Biobank, whose population is homogenous. The identified SNPs must be validated on more diverse patient populations before being translated towards clinical utility. Future work should consider the development of multi-modal models with an expanded and diverse patient cohort from multiple sites. These models should consider other clinically relevant outcomes that may be well predicted by patient imaging and notes in ASD. Future models that seek to predict actionable clinical outcomes should define outcomes considering important parameters which impact the clinical outcome (i.e., fusion rate, screw malposition, screw breakage, and amount of correction) [30].

## 5. Conclusions

This study demonstrates that, while predictive efforts in neurosurgery are often limited by available patient samples, complex models (CNN, LLM, GWAS) offer predictive potential for surgical outcome prediction. These models, which incorporate more powerful machine learning technologies, can improve the clinical workflow by the elimination of manual input of patient features and the inclusion of patient images and clinical notes for prediction. The models can be developed in future work to robustly predict complications following surgical intervention for adult spinal deformity, improving patient selection and counseling.

## Figures and Tables

**Table 1:** Performance metrics for the CNN trained from scratch (top) and the fine-tuned CNN from VinDr-SpineXR pre-training (bottom). A separate CNN was trained for each outcome. Area under the curve (AUC), F1 score, precision, sensitivity, and specificity metrics are included.

**CNN**	**AUC**	**F1**	**Precision**	**Sensitivity**	**Specificity**
Pulmonary Complication	0.579	0.288	0.235	0.326	0.822
Neurological Complication	0.619	0.224	0.137	0.447	0.766
Sepsis	0.534	0.132	0.066	0.198	0.917
Delirium	0.654	0.168	0.092	0.478	0.756
**Pre-Trained CNN**	**AUC**	**F1**	**Precision**	**Sensitivity**	**Specificity**
Pulmonary Complication	0.552	0.259	0.212	0.290	0.819
Neurological Complication	0.545	0.169	0.092	0.620	0.491
Sepsis	0.638	0.135	0.069	0.713	0.504
Delirium	0.414	0.000	0.000	1.000	0.000

**Table 2:** Performance metrics for the NLP models that were separately trained for each outcome. The NLP model is a fine-tuned Gatortron LLM. Area under the curve (AUC), F1 score, precision, sensitivity, and specificity metrics are included.

LLM	AUC	F1	Precision	Sensitivity	Specificity
Pulmonary Complication	0.565	0.545	0.410	0.814	0.317
Neurological Complication	0.559	0.250	0.467	0.171	0.946
Sepsis	0.557	0.383	0.440	0.338	0.776
Delirium	0.508	0.156	0.085	1.000	0.017

**Table 3:** GWAS results. Significant single nucleotide polymorphisms (SNPs) below a 10 × 10^−5^ threshold are included. SNPs are detailed according to their location, reference allele, alternate allele, standard rsID, and mapped to their respective gene by rsID.

Chromosome	SNP	rsID	Reference Allele	All Alternate Alleles	OR	*p*	Gene	Coding Variant	Clinical Significance
1	1:44000938:G:A	rs76038188	G	A	2.77567	5.3 × 10^−6^	SLC6A9	No	Benign
1	1:44001675:C:T	rs188509294	C	T	4.31614	7.2 × 10^−7^	SLC6A9	No	
1	1:44002768:C:T	rs41270407	C	T	2.80768	4.2 × 10^−6^	SLC6A9	No	Benign
1	1:44010680:G:T	rs62621784	G	T	2.81042	4.1 × 10^−6^	SLC6A9	No	Benign
1	1:155612177:T:C	rs470548	T	C	7.41008	2.9 × 10^−7^	MSTO1	No	Benign
2	2:175964654:C:A	rs144765990	C	A	3.94093	2.8 × 10^−6^	LNPK	No	
4	4:16674160:C:A	rs191683804	C	A	8.05679	2.3 × 10^−6^	LDB2	No	
5	5:138150951:A:T	rs79552163	A	T	2.36997	5.4 × 10^−6^	BRD8	Yes	
6	6:53097675:G:A	rs9474385	G	A	3.52686	3.9 × 10^−6^	FBXO9	No	
6	6:64626250:G:A	rs9445051	G	A	1.57604	4.2 × 10^−6^	EYS	No	Benign
10	10:103406519:A:G	rs17735658	A	G	3.87312	1.3 × 10^−7^	PDCD11	No	
15	15:42737116:C:T	rs190314153	C	T	5.62477	6.5 × 10^−6^	CDAN1	No	Uncertain significance, Benign
22	22:39647872:C:A	rs57732048	C	A	4.384	7.2 × 10^−6^	CACNA1I	Yes	
22	22:46365600:C:G	rs12165943	C	G	1.58045	4.4 × 10^−6^	CELSR1	Yes	
22	22:46373108:T:C	rs6008779	T	C	1.64463	6.9 × 10^−6^	CELSR1	No	
22	22:46378549:C:G	rs56344079	C	G	1.68868	2.3 × 10^−6^	CELSR1	No	
22	22:46384624:T:C	rs6007897	T	C	1.69137	1.1 × 10^−6^	CELSR1	Yes	
22	22:46391537:C:T	rs6008793	C	T	1.65223	3.8 × 10^−6^	CELSR1	No	
22	22:46391797:A:G	rs6008794	A	G	1.64112	5.0 × 10^−6^	CELSR1	Yes	
22	22:46391800:A:G	rs6008795	A	G	1.63249	6.7 × 10^−6^	CELSR1	Yes	
22	22:46394132:G:T	rs11703679	G	T	1.6524	3.5 × 10^−6^	CELSR1	No	

## Data Availability

Publicly available datasets were analyzed in this study. This data can be found here: https://vindr.ai/datasets/spinexr (accessed on 18 December 2023). Restrictions apply to the availability of some data analyzed in this study. Data was obtained from Stanford Research Repository and are under IRB and Stanford University restrictions.

## Supplementary Materials

The following supporting information can be downloaded at: https://www.mdpi.com/article/10.3390/jcm13030656/s1, Figure S1: Loss curves for training (blue) and validation (red) cohorts for NLP model training on (a) pulmonary complications, (b) neurological complications, (c) sepsis, (d) delirium. The NLP models begin to overfit to the training set after 10 epochs of fine tuning across the four outcomes.
