# Supplementary figures and images for: Evaluating Computer Vision, Large Language, and Genome-Wide Association Models in a Limited Sized Patient Cohort for Pre-Operative Risk Stratification in Adult Spinal Deformity Surgery
Source: J Clin Med. 2024 Jan 23;13(3):656. doi: 10.3390/jcm13030656 (PMC10856542; doi:10.3390/jcm13030656)

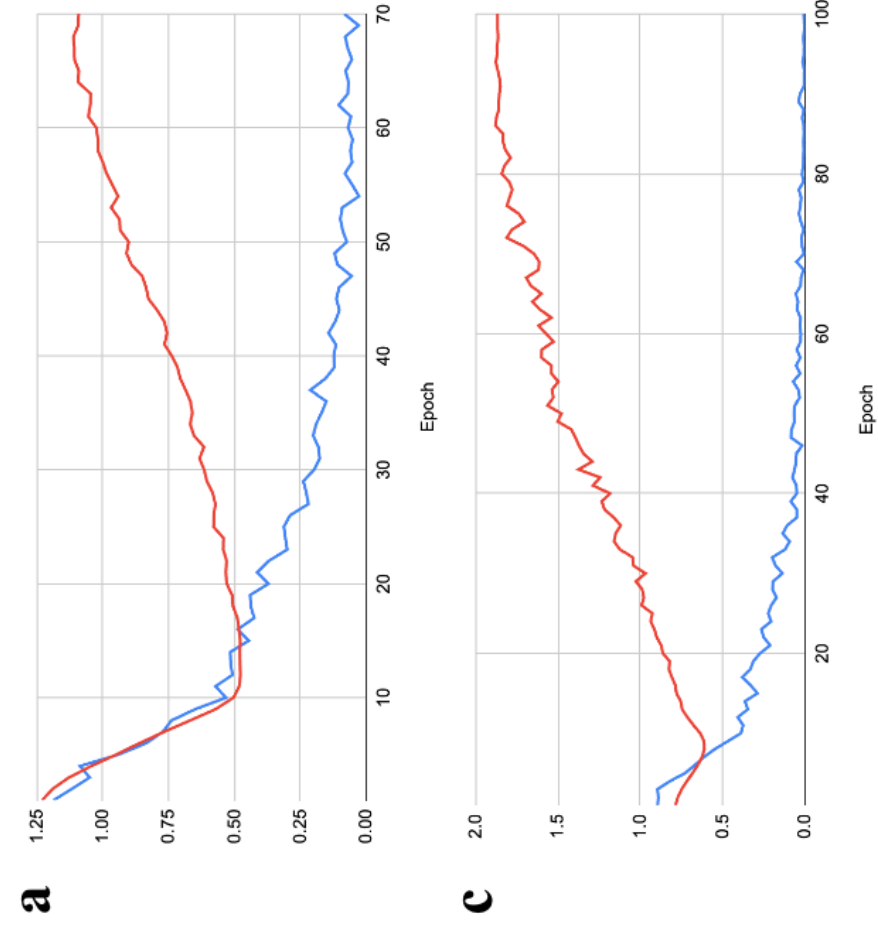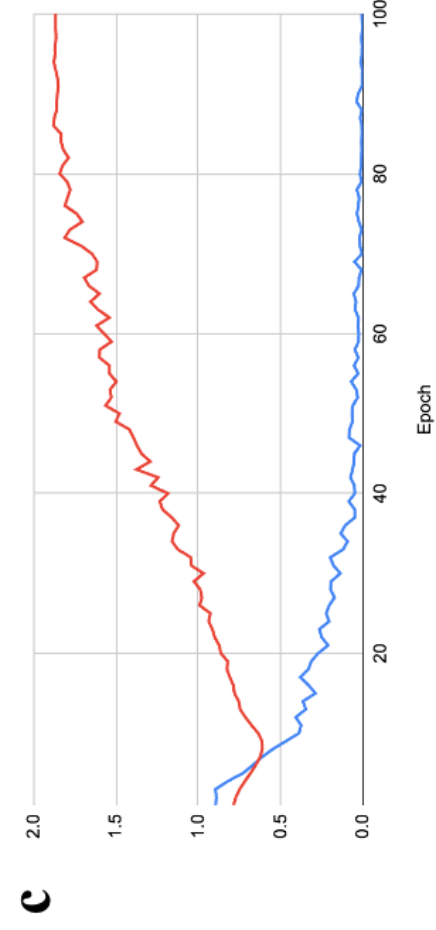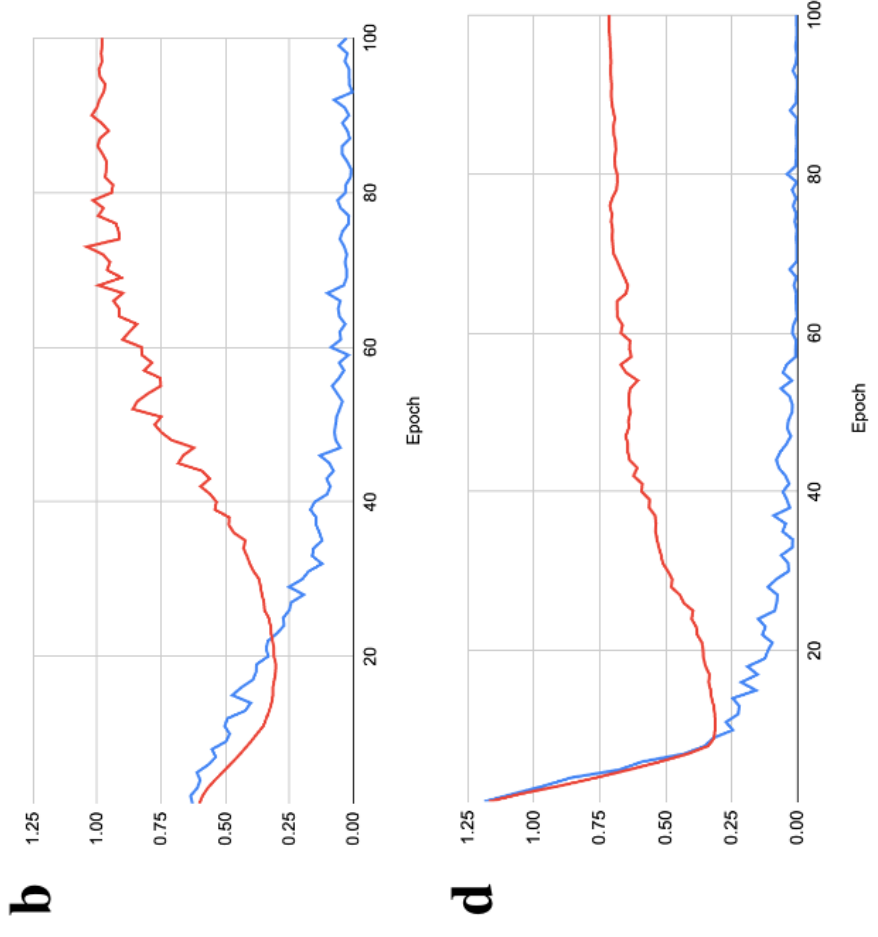

Supplement: Supplementary file 1 [file jcm-13-00656-s001.zip › jcm-2806379-Figure S1.pdf]
